# Structure and Dynamics of the G121V Dihydrofolate Reductase Mutant: Lessons from a Transition-State Inhibitor Complex

**DOI:** 10.1371/journal.pone.0033252

**Published:** 2012-03-13

**Authors:** Randall V. Mauldin, Paul J. Sapienza, Chad M. Petit, Andrew L. Lee

**Affiliations:** 1 Department of Biochemistry and Biophysics, School of Medicine, University of North Carolina at Chapel Hill, Chapel Hill, North Carolina, United States of America; 2 Division of Chemical Biology and Medicinal Chemistry, Eshelman School of Pharmacy, University of North Carolina at Chapel Hill, Chapel Hill, North Carolina, United States of America; The Scripps Research Institute, United States of America

## Abstract

It is well known that enzyme flexibility is critical for function. This is due to the observation that the rates of intramolecular enzyme motions are often matched to the rates of intermolecular events such as substrate binding and product release. Beyond this role in progression through the reaction cycle, it has been suggested that enzyme dynamics may also promote the chemical step itself. Dihydrofolate reductase (DHFR) is a model enzyme for which dynamics have been proposed to aid in both substrate flux and catalysis. The G121V mutant of DHFR is a well studied form that exhibits a severe reduction in the rate of hydride transfer yet there remains dispute as to whether this defect is caused by altered structure, dynamics, or both. Here we address this by presenting an NMR study of the G121V mutant bound to reduced cofactor and the transition state inhibitor, methotrexate. NMR chemical shift markers demonstrate that this form predominantly adopts the closed conformation thereby allowing us to provide the first glimpse into the dynamics of a catalytically relevant complex. Based on ^15^N and ^2^H NMR spin relaxation, we find that the mutant complex has modest changes in ps-ns flexibility with most affected residues residing in the distal adenosine binding domain rather than the active site. Thus, aberrant ps-ns dynamics are likely not the main contributor to the decreased catalytic rate. The most dramatic effect of the mutation involves changes in µs-ms dynamics of the F-G and Met20 loops. Whereas loop motion is quenched in the wild type transition state inhibitor complex, the F-G and Met20 loops undergo excursions from the closed conformation in the mutant complex. These excursions serve to decrease the population of conformers having the correct active site configuration, thus providing an explanation for the G121V catalytic defect.

## Introduction

High resolution models of enzymes bound to substrate and transition-state analogs have provided keen insight into the power of biological catalysts. These models reveal that enzymes lower the activation barrier for chemistry relative to the analogous solution reactions by fixing the positions of catalytically important atoms and shielding reactive groups from bulk solvent [1]. Simple inspection of the architecture of these complexes shows that pre-organization is not the whole story and that enzymes must be flexible to accommodate bond making and breaking as well as binding and release of reactants and products. Indeed, with the advent of NMR spectroscopic tools, it has been shown that enzyme dynamics help to shepherd reactants and products through energetically rugged reaction coordinates [2], [3], [4], [5]. In addition to a role in substrate flux, it has been proposed that enzyme fluctuations may also promote the chemical step itself [6], [7], [8] although this assertion remains controversial [9], [10].

**Figure 1:**
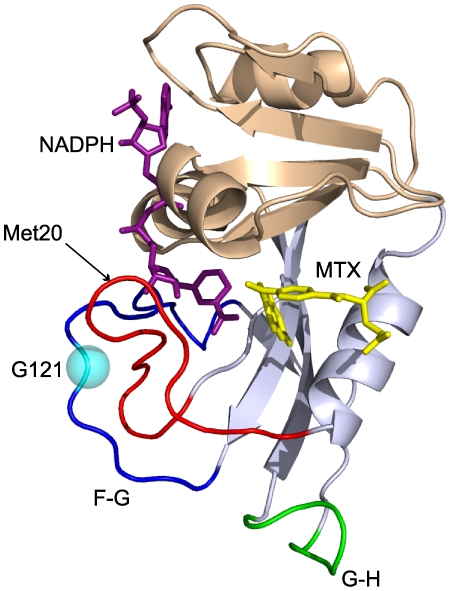
DHFR subdomain and loop nomenclature. Structure of DHFR complexed with NADPH (purple) and methotrexate (yellow) (pdbid 1RX3). Adenosine binding domain is shown in light blue while the loops domain is shown in wheat. The Met20 (red), F–G (blue), G–H (green) loops are labeled. The site of mutation (G121V) is indicated by a cyan sphere.

Dihydrofolate reductase (DHFR) is among the most highly studied enzymes from the standpoint of flexibility. The active site of DHFR is surrounded by the Met20, F–G and G–H loops (Figure 1); Wright and co-workers have shown that the dynamic motions of these loops play a functional role in the catalytic cycle [2]. Motions in the loops have also been implicated in promoting catalysis using both theoretical [11], [12], and experimental [6], [13], [14] techniques. Indeed, mutations within the Met20 [15], F–G [16], or G–H loops [17] “allosterically” modulate DHFR catalysis. Thus, the interactions within the loops surrounding the active site are uniquely coupled to function. Glycine 121 (G121) is one of the most storied points of mutation in DHFR. G121 is located in the F-G loop (Figure 1) and is highly conserved [18]. Substituting valine or leucine in place of G121 decreases the rate of steady-state catalysis (*k*_cat_) 20-fold [19] despite being ∼15 Å from the active site. The analysis of the complete kinetic scheme showed that G121V reduces the rate of chemistry (hydride transfer) by 170-fold making hydride transfer, rather than product release as is the case with the wild type, the rate limiting step [13]. Although dissection of G121V kinetics has yielded insights into the effect of the mutation, questions remain about how a mutation on a distal loop severely impairs chemistry at the active site. On one hand, there is clearly a steric component to the effect of substitution because *k*_hyd_ follows the hierarchy: Gly>Ala>Ser>Val>Leu [11], [13]. This structural hypothesis is supported by kinetic isotope effect studies [14] and by simulations of the E^G121V^:NADP+:folate complex, showing that the G121V mutation interferes with contacts between the F-G and Met20 loops; this causes differences in co-factor substrate orientation and results in fewer near attack conformers [20]. Further, Wright and coworkers have reported that DHFR G121V adopts the occluded rather than the closed conformation in the E^G121V^:NADP+:folate complex, raising the possibility that the catalytic defect arises from a failure to close the Met20 loop around the active site [21]. It should be noted that this study used NMR chemical shifts from alanine residues only [21]. On the other hand, others have suggested a dynamic hypothesis in which the G121V substitution interrupts a “network of coupled promoting vibrations” that are required for DHFR catalysis [13], [18]. Indeed, our work showed that the active site of DHFR is dynamically coupled to G121. When methotrexate (MTX) is added to the DHFR holoenzyme forming the ternary E:NADPH:MTX complex, G121 becomes more flexible on the ps-ns timescale and chemical exchange on the µs-ms timescale is quenched [22]. Thus, motion within the F–G loop can be controlled by interactions within the active site approximately 15 Å away.

Here we use NMR probes into structure and dynamics to understand whether the catalytic defect resulting from G121V mutation arises from changes in structure, flexibility, or both. We show the enzyme can be isolated in the closed conformation in complex with NADPH and the transition-state analog MTX. By studying the mutant bound to the reduced cofactor and a transition-state analog, we take the first step towards understanding the dynamics of G121V in a catalytically relevant conformation. We find a limited ps-ns timescale dynamic response to DHFR G121V at the active site based on backbone and side-chain relaxation techniques. In fact, the most widespread changes to dynamics caused by the mutation are not in the active site but rather in the distal adenosine binding domain. The most striking effect is a result of destabilization of the closed F–G and G–H loop conformations as evidenced by increased µs-ms dynamics relative to the wild type enzyme. Thus, our results support the hypothesis that sub-nanosecond dynamics are not the main contributor to the altered rate of catalysis in DHFR G121V. Rather, altered dynamics of the F-G and Met20 loops on the µs-ms timescale indicate more frequent excursions from the closed complex and hence a lower population of conformers that are catalytically competent.

## Results

### A high affinity drug traps G121V DHFR in the catalytically relevant ‘closed’ conformation

**Figure 2:**
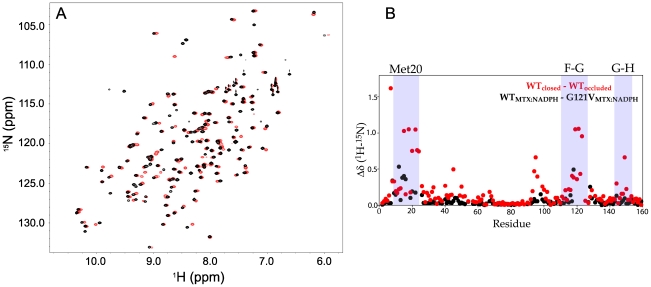
Methotrexate binding traps G121V in the closed conformation. (A) ^1^H–^15^N HSQC spectra of E:NADPH:MTX (black) overlain with the ^1^H–^15^N HSQC spectra of E^G121V^:NADPH:MTX (red). (B) The reduced change in chemical shift is calculated using the following formula and is subsequently plotted as a function of residue number: 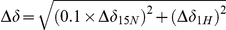. The changes as a result of mutation (G121V vs. WT) are shown in black and a bona fide closed–occluded change are plotted in red [23]. The plot shows that the pattern of changes as a result of mutation can be attributed to differences in the local chemical environment and not large-scale structural change. The Met20 (residues 9–23), F–G (residues 117–131), and G–H (residues 142–149) loops are highlighted.

The catalytic step of hydride transfer occurs in the complex with the DHFR Met20 loop in the closed conformation [2]. The goal of this study was to examine the dynamics of DHFR G121V in the closed conformation in an effort to provide insights into the origins of the substantial decrease in hydride transfer rate. Chemical shift analysis indicated that DHFR G121V does not readily adopt the closed conformational ensemble. Ligands such as NADP+ and folate stabilize the closed ensemble of WT DHFR, whereas G121V is occluded in this complex [21]. However, based on our previous finding that DHFR is “locked” into the closed conformation by high affinity inhibitors [22], we sought to investigate the structure of DHFR G121V in complex with NADPH and the transition-state analog, MTX. We used backbone chemical shifts as structural probes since these are sensitive to the conformational state of DHFR [23]. Chemical shifts were assigned using standard triple resonance experiments (see methods). A vast majority of the resonances could be assigned with the notable exception of the F-G loop. Assignments for residues 117 and 118 were complicated due to extensive exchange broadening and 119–125 were missing. Resonances from each of these residues were present in wild type spectra. It is likely that increased relaxation due to conformational exchange resulted in extensive line broadening in the mutant assignment spectra. This hypothesis is supported by exchange broadening of resonances in the Met20 loop (residues 13–16) that directly contact the F-G loop. In all, 129 out of 148 non-proline residues could be assigned. As shown in Figure 2A, the HSQC spectra of WT and G121V DHFR bound to NADPH and MTX are nearly identical. A quantitative analysis of chemical shift changes resulting from the mutation is shown in Figure 2B. As anticipated, the largest changes in chemical shift are observed for residues 12–16 and 118, which are near the site of mutation. It should be noted that a hydrogen bond between the amide hydrogen of D122 and the carbonyl oxygen of G15 is involved in stabilization of the closed state, and the G121 mutation causes a perturbation in the chemical shift of G15 (Figure 2B). (The other partner in this hydrogen bond, D122, could not be assigned in the mutant due to exchange broadening.) However, due to proximity of these residues to the site of mutation it is not possible to de-convolute an effect from potential disruption of this hydrogen bond from the effect of the mutation itself. It is therefore instructive to look at the chemical shifts of residues 7, 93–96, and 148, since they are diagnostic of the closed vs. occluded status of DHFR and distal to residue 121 [23]. These residues experience conformation dependent changes in their amide hydrogen bond interactions and thus serve as markers for the Met20 loop structure. Alanine 7 forms a hydrogen bond with the nicotinamide ring of NADPH in the closed conformation. This interaction cannot occur in the occluded complex because the side chain of M16 occludes the active site. The chemical shifts of residues 93–96 are also sensitive to the nicotinamide ring of NADPH moving out of the active side, which occurs upon the closed to occluded transition [23]. Lastly, there is a hydrogen bond between the amide group of S148 and the carbonyl group of N23 in both the open and occluded states, but not the closed conformation [24]. We do not observe changes in chemical shifts at any of these marker residues (Figure 2B) indicating that DHFR G121V adopts a conformational ensemble that resembles the WT closed complex. By comparison, if we examine the changes in chemical shift between *bona fide* closed and occluded complexes, the changes are quite drastic, and much larger than the modest changes as a result of mutation (Figure 2B). We therefore conclude from chemical shift analysis that while bound to MTX and NADPH, DHFR G121V predominantly adopts a closed conformation that is a good representation of a catalytically relevant complex.

### G121V mutation induces aberrant conformational exchange in the Met20 and F-G loops

**Figure 3:**
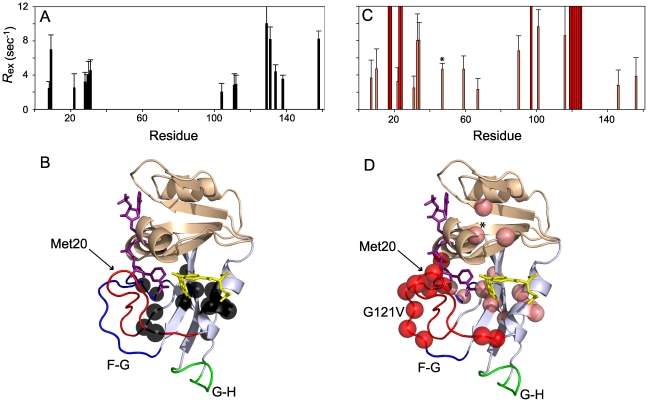
G121V dependent changes in µs-ms motions. Backbone residues with significant (>1.5σ) *R*_ex_, as determined by ^15^N CPMG relaxation experiments for E:NADPH:MTX (A) and E^G121V^:NADPH:MTX (C). For E^G121V^:NADPH:MTX, residues with calculated exchange rates are in pink while residues that could not be assigned because of line broadening are in red. Common residues that could not be assigned in either ternary complex are not included. These residues also mapped onto the structure using black spheres for E:NADPH:MTX (B) and pink or red spheres, denoting CPMG-based *R*_ex_ or missing respectively, for E^G121V^:NADPH:MTX (D).

It is well established that conformational switching on the µs-ms timescale plays a role in progression through the catalytic cycle in DHFR [2] and other enzymes [3], [4]. In DHFR, the E:NADP+:folate complex, which is a model for the Michaelis complex, switches between a major state in the closed conformation and a minor occluded state that structurally resembles the next step in the catalytic cycle [2], [25]. This global switching event involves substrate binding residues as well as the F-G and Met20 loops. We have recently shown that the E:NADPH:MTX complex adopts the closed complex; however, drug binding affects the distribution of residues undergoing motion on the µs-ms timescale [22]. While the drug bound enzyme still undergoes the same switching event in the active site as the E:NADP+:folate complex, motions in the F-G and Met20 loops are quenched [22]. Does MTX binding to the G121V mutant elicit the same dynamic response? Our observation that multiple resonances from the F-G and Met20 loops are broadened or absent from HSQC spectra suggests that MTX binding to the G121V mutant is unable to quench µs-ms motions of these loops (Figure 3). In addition to the strong inference of chemical exchange based on missing peaks, we also measured the excess exchange contribution (*R*_ex_) to the overall transverse relaxation rate (*R*_2_) for observable resonances using relaxation compensated CPMG experiments [26]. By measuring the effective ^15^N *R*_2_ rates at two CPMG field strengths, one low, to retain the effects of exchange, and one high to minimize the effects of exchange, an estimate of *R*_ex_ can be calculated from the simple difference between the two *R*_2_ rates. Both datasets were acquired at the same magnetic field strength (500 MHz ^1^H frequency) to ensure comparability. In this analysis we assigned significant *R*_ex_ to residues having a greater than 2 Hz difference in *R*_2_ with the difference being greater than 1.5-times the propagated error. Because the enzyme is greater than 99.9% saturated with both NADPH and MTX under these experimental conditions, *R*_ex_ is manifest from *intra*molecular motions and not binding/release of ligands (see Materials and Methods). From this experiment, we find significant overlap in the regions of DHFR that undergo chemical exchange in the wild type and E^G121V^:NADPH:MTX complexes (Figure 3). There are some residues with significant *R*_ex_ in the wild type but not the mutant (residues 8, 9, 22, 104, and 111), but in most of these cases, mutant resonances are broadened and errors in *R*_2_ prevented precise calculation of *R*_ex_. Further, in all of these cases, there are mutant residues with significant chemical exchange nearby (Figure 3A&C). Taken together, the *R*_ex_ measurements and location of regions with missing or broadened resonances show that the G121V mutant has a chemical exchange profile that more closely resembles the wild type E:NADPH [22] or E:NADP+:folate [2] complexes with exchange in the Met20 loop, the F-G loop, and substrate-binding residues, than the wild type E:NADPH:MTX complex where exchange is limited to substrate binding residues [22] (Figure 3B&D). Lastly, resonances from R52 and I61 exhibit significant *R*_ex_ in the mutant, but not the wild type. In support of this, “model-free analysis” of the W47 indole indicates a greater amount of chemical exchange in G121V than the wild type (see below). The side chain of W47 is packed between R52 and I61 (Figure 3D). These residues, within the adenosine binding domain, reside in structural elements that undergo chemical exchange in a number of occluded DHFR forms [2].

### G121V mutation has limited effect on ps-ns timescale flexibility of the active site

One explanation for the catalytic defect caused by the G121V mutation involves disruption of motions on a broad range of timescales (fs-ms) that are coupled to catalysis [18]. To measure the effect of the mutation on sub-ns fluctuations, we performed a series of NMR spin relaxation experiments. Backbone dynamics on the ps-ns timescale of the E^G121V^:NADPH:MTX complex were probed by measuring ^15^N *R*_1_, ^15^N *R*_2_, and {^1^H}-^15^N NOE at two magnetic fields (500 and 600 MHz ^1^H) and analyzed using the Lipari Szabo model-free formalism [27], [28]. From this, we obtained order parameters (*S* ^2^), which describe the amplitude of motion, and internal correlation times (τ_e_), describing the timescale of motion for each non-proline backbone N-H bond vector. Order parameters range from 0 to 1 representing isotropic motion and fixed orientation of bond vectors respectively. The global tumbling time (τ_m_) is required for this analysis and the G121V ternary complex τ_m_ was found to be 10.7 ns/rad, which is comparable to 10.5 ns/rad for the wild type ternary complex [22]. AIC statistics were used to determine the best model to describe backbone motion [29].

**Figure 4:**
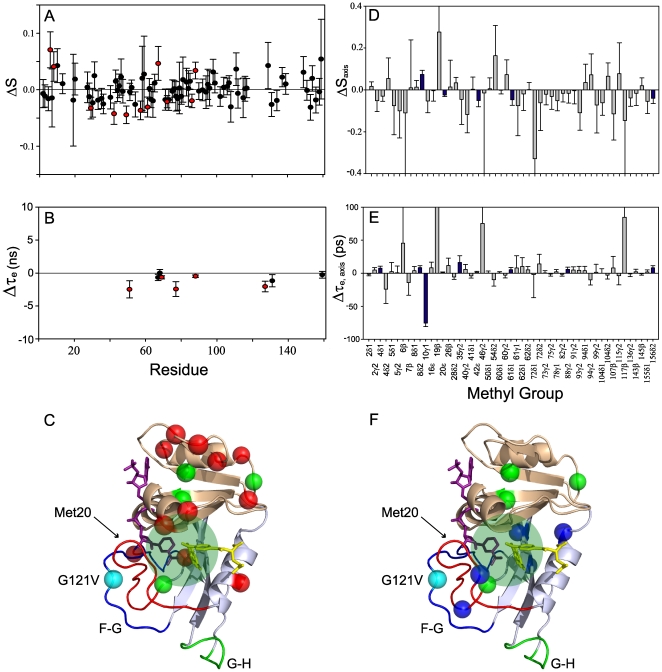
G121V dependent changes in backbone and side-chain dynamics on the ps-ns timescale. Changes in backbone *S* ^2^ (A) and τ_e_ (B) order parameters determined from ^15^N relaxation experiments. Changes in side-chain *S* ^2^_axis_ (D) and τ_e,__axis_ (E) order parameters determined from ^2^H relaxation experiments. Plotted values were obtained by subtracting mutant (E^G121V^:NADPH:MTX) from wild-type (E:NADPH:MTX) parameters with significant changes (>1.5σ) highlighted in red (backbone) and blue (side-chain). Significant changes are also mapped onto the structure using red spheres for backbone (C) and blue spheres for side-chain (F). Greens spheres denote residues that have significant changes in both backbone and side-chain order parameters. An area of 5 Å around the active site is highlighted in green.

The changes in backbone order parameters resulting from the G121V substitution (E^G121V^:NADPH:MTX complex) are shown in Figure 4. The residues showing significant changes in dynamics (difference in *S* ^2^ *or* τ_e_ greater than 1.5*σ) are 6, 8, 29, 42, 49, 51, 57, 61, 67, 69, 72, 77, 86, 88 and 127. These residues are mapped onto the E:NADPH:MTX structure in Figure 4C. With the exception of 6, 8, 29, and 127, the residues with perturbed backbone dynamics are in the adenosine binding domain and thus distal to the site of mutation (Figure 4C). Further, A6 is the only residue with significantly perturbed dynamics within 6 Å of the site of hydride transfer (Figure 4C); this is a surprising observation given the dramatic effect this mutation has on the rate of the chemical step. Finally, while the mutation results in a modest increase in flexibility for most of the residues, the backbone at positions 6, 8, 67, and 88 becomes more rigid in the mutant.

Because side-chain dynamics are often more responsive to perturbation than the main-chain [30], we sought to measure the effect of the G121V mutation on DHFR methyl dynamics. We performed ^2^H qaudrupolar *D*_z_ and *D*_y_ relaxation [31], [32] on E:NADP:MTX complexes in which DHFR is selectively enriched with CH_2_D isotopomers. The measurements on the wild type complex were reported previously [22]. To minimize peak overlap, the relaxation datasets were collected at 600 and 700 MHz field strength. We noticed that the signal-to-noise of the G121V *D*_z_ and *D*_y_ spectra were lower than the wild type counterparts, presumably due to increased conformational exchange (see above). This prompted us to acquire the mutant data at 2 mM enzyme instead of on 1 mM samples that were used for other NMR experiments. Methyl symmetry axis order parameters (*S* ^2^_axis_) and internal correlation times (τ_e,axis_) were determined by simultaneously fitting the *D*_z_ and *D*_y_ rates at two fields to the model-free spectral density function. A total of 54 out of 92 methyl groups could be measured for this mutant and we were able to compare 51 methyl groups between the wild type and E^G121V^:NADPH:MTX complexes. The changes in *S* ^2^_axis_ and τ_e,axis_ are shown in Figure 4D&E. Significant differences in *S* ^2^_axis_ and τ_e,axis_ are restricted to 4^Cδ1^, 8^Cδ2^, 10^Cγ1^ 20^Cε^, 35^Cγ2^, 42^Cε^, 61^Cδ1^, 88^Cγ2^, and 156^Cδ2^. In comparison with different mutations in DHFR [33] and other systems [34], [35], the effect of the G121V mutation on side-chain dynamics is rather limited. This could be a real feature of this mutant or a result of the higher noise inherent in this dataset. We favor the former explanation since the average value of Δ*S* ^2^_axis_ for residues without statistically significant change is very close to zero (−0.02), which indicates random noise in the data. Of the residues with significantly altered dynamics, three lie in the loops domain and four lie in the adenosine binding domain. Of these, only M20 is within 6 Å of the site of hydride transfer (Figure 4F). Interestingly, residues 8, 42, and 61 have significant dynamical perturbation to both the backbone and methyl side-chain. The direction of the perturbation matches for both bond vectors in that the main chain and side chains of residue 8 become more rigid, while 42 and 61 become more flexible (Figure 4A&D).

DHFR has five tryptophan residues that provide additional insight into side-chain dynamics. In both the wild type and E^G121V^:NADPH:MTX complexes, the relaxation rates of each tryptophan could be measured using the standard relaxation experiments. The model-free analysis of dynamics of tryptophan N^ε^-H bond vectors for both the wild type and mutant complex are summarized in Table 1. In general, the dynamics of the tryptophan side chains do not change, with the notable exception of W22, which rigidifies in the mutant, and W47, which has a larger *R*_ex_ component than the wild type.

**Table 1:** Model-free analysis of tryptophan indole ^15^N relaxation within E:NADPH:MTX and E^G121V^:NADPH:MTX complexes.

Residue	*S* ^2^	*S* ^2^_err_	τ_e_ (ps)	τ_e,err_	*R*_ex_ (s^−1^)^a^	*R*_ex,err_	χ^2^
**WT**							
22N^ε^	0.91	0.01			1.44	0.20	49.9
30N^ε^	0.94	0.02					13.3
47N^ε^	0.86	0.01	12.3	6.2	1.50	0.22	37.9
74N^ε^	0.97	0.01					17.7
133N^ε^	0.93	0.01					43
**G121V**							
22N^ε^	0.97	0.04			0.76	0.65	7.86
30N^ε^	1.00	0.04	111.0	345.0	0.71	0.28	1.95
47N^ε^	0.86	0.02			4.68	0.68	3.16
74N^ε^	0.96	0.02	38.0	22.3	1.26	0.34	14.2
133N^ε^	0.92	0.02	6.6	7.6	1.43	0.31	5.65

a *R*_ex_ values are based on model-free fits.

## Discussion

### Reduced cofactor and transition-state analog trap G121V in the closed conformation

DHFR catalyzes the NADPH-dependent reduction of DHF to THF. For this reaction to take place, the enzyme must adopt the “closed” conformation in which the nicotinamide moiety of NADPH occupies the active site. Although previous studies have shown that the G121V enzyme is not in the closed conformation when bound to any ligands [21], we demonstrate here, using chemical shift markers, that E^G121V^:NADPH:MTX is a closed complex. Our results do not contradict earlier work on the mutant since Venkitakrishnan et al. did not use either reduced cofactor or a transition state analog [21]. It is important to note here that MTX cannot be regarded as a perfect transition-state analog because the pteridine ring of MTX is flipped 180° relative to that of folate [24]. However, several features of the E:NADPH:MTX complex suggest this complex most resembles the transition state: 1) MTX binds with ultra high affinity (*K*_D_  =  27 pM [36]) as is predicted for enzyme-transition state inhibitor complexes, 2) The E:NADPH:MTX complex is characterized by increased closure of the pABG binding cleft relative to the E:NADP+:FOL model of the Michaelis complex. This allows for sub van der Waals juxtaposition of hydride donor and acceptor and an increase in active site hydrophobicity, both of which are required in the transition state [24], [37]. Taken together, these observations support our assertion that the E^G121V^:NADPH:MTX complex is the best model for examining the dynamics in a conformation that is poised for catalysis.

### Altered ps-ns dynamics are not the lone origin of the G121V catalytic defect

In this work we show that the backbone amide and side-chain methyl ps-ns dynamics of wild type and G121V DHFR are generally very similar when bound to NADPH and MTX . This observation is consistent with the mutant and wild type protein complexes adopting the closed conformation in solution. We do note small, but significant dynamical perturbation at residues known to be important for function and protein stability. For example, both the amide and methyl group of M42 exhibit altered dynamics in the mutant. Previous kinetics work suggests M42 and G121 are “linked”, in that the mutations show non-additivity in their effects on reaction kinetics and structure [14]. Here, we show mutation at the residues are dynamically coupled in that G121 slightly alters the dynamics of M42. Similarly, the G121V mutation also affects dynamics at G67, which is ∼25 Å away. These two positions were shown to be coupled based on non-additive effects on stability and *k*_cat_ [38]. Finally, there are also small, but significant changes in the pABG binding cleft (S49 and R57), and I61 in the adenosine binding domain. We have shown that the dynamics of I61 is sensitive to ligand binding [22], the M42W [33] substitution, and now the G121 substitution. Furthermore, I61 is highly conserved in DHFR and is potentially coupled to the active site [39]. Interestingly, all the residues mentioned above, and the majority of all the significant ps-ns dynamic responders lie in the adenosine binding domain. Taken together, these results support the hypothesis that long range thermodynamic couplings are the result of altered dynamic modes within the ground state of proteins [40] rather than direct coupling to the chemical step itself (see below).

If changes in ps-ns dynamic did play a large role in reducing the rate of hydride transfer, then we would expect perturbations to be detected by residues in and around the active site. The mutation does cause a handful of changes to ps-ns dynamics in the loops domain; these changes are limited to A6, L8, M20, A29, and D127. Could changes in ps-ns motion be responsible for the severe impairment in G121V catalyzed hydride transfer? It was proposed by Hammes-Schiffer and colleagues that G121V interrupts a network of coupled promoting motions that serve to aid catalysis [18]. In the wild type enzyme, the residues involved in this network are I14, G15, F31, M42, Y100, G121 and D122 [11]. Hybrid QM/MM simulations show that motions between F31 and NADPH, G15 and D122, and I14 and NADPH are uncoupled from the reaction coordinate in the G121V mutant [18]. Of the putative catalysis promoting network, M42 is the only residue whose ps-ns dynamics are affected by the G121V mutation. However, we emphasize that resonances from I14 and I15 showed severe line-broadening and therefore large uncertainty in model-free parameters, and V121 and D122 were completely missing from spectra (also due to chemical exchange line-broadening). While changes in the µs-ms timescale prevent direct measurement of the ps-ns dynamics, it is possible that changes on the µs-ms timescale are a reflection of ps-ns perturbations [41]. Finally, recent work using quantized molecular dynamics simulations reproduced the statistical correlations between participants in the putative wild type network [42]. In contrast to previous work, dynamical correlations (defined as “coupling of inertial atomic motions”) that are coupled to the hydride transfer step were found to be limited to a sphere of 4–6 Å from the donor-acceptor pair [42]. Motions outside of this sphere were completely uncoupled from the hydride transfer event. Furthermore, the fluctuations that guided the system to the transition state took place on the timescale of femtoseconds [42]. Dynamics on the fs timescale do influence the ^15^N and ^2^H relaxation rates measured here [27]; however, a limitation of model-free analysis is that it cannot de-convolute the contribution of fluctuations at discrete frequencies. Changes in model-free parameters do inform us that there are differences in dynamics at frequencies higher than overall tumbling (∼10 ns) and as described above, we observe two groups (A6 and M20) whose sub-ns dynamics are perturbed by G121V *and* are within 6 Å of the reactive center (Figure 4C&F). While the overall changes in protein dynamics are small, our data support the hypothesis that changes in motional modes may contribute in some way to decreasing the catalytic rate. However, given the limited dynamic response it is likely that changes is ps-ns dynamics are not the sole contributor to the drastic catalytic defect observed in this mutant protein.

### Novel excited state in the E^G121V^:NADPH:MTX complex

CPMG relaxation dispersion experiments show that the wild type and E^G121V^:NADPH:MTX complexes share some common features in their µs-ms flexibility profiles. Both complexes exhibit chemical exchange around the active site that was described previously [22]. However, initial inspection of the G121V HSQC spectra indicates several resonances are severely weakened due to exchange broadening. As described above, residues 13–16 are very weak in the HSQC spectrum and residues 119–126 and 17–18 could not be assigned because they are missing. These results indicate that MTX binding to the E^G121V^:NADPH complex is unable to quench motions within the Met20 and F–G loops. The degree of line broadening from chemical exchange (*R*_ex_) is determined by the rate of exchange (*k*_ex_) , the chemical shift difference between the two states (Δω), and the population of the minor state (p_B_) (equation 1).
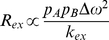
(1)For resonances that are completely absent, it is not possible to parse out the relative contributors to the large *R*_ex_. Resonances are broadened for residues adjacent to those that are missing. However, sensitivity issues prevent us from extracting parameters from full CPMG experiments. Nonetheless, it is clear that the two loops in the mutant complex are undergoing increased dynamics relative to the wild type transition state complex. Because the chemical shifts of the two complexes are very similar, we favor a hypothesis where a high population of the excited state (p_B_) contributes to the large *R*_ex_. These increased dynamics are likely a result of destabilized hydrogen bond interactions between the Met20 and the F–G loops due to steric clash with the bulky valine side-chain. DHFR G121V may be transiently switching between the closed and occluded conformations. However, this seems unlikely because we do not observe any increased µs-ms dynamics in the G–H loop. A more probable explanation is that the nicotinamide moiety of NADPH is “flipping” into and out of the active site. These results support the hypothesis that the 3.5 s*−*1 pre-chemistry structural rearrangement reflects the nicotinamide moiety of NADPH entering the active site [13].

It is interesting to consider our results in the context of a recent study on the influence of µs-ms dynamics of the chemical step in DHFR. Wright and colleagues found that a proline insertion at position 23 coupled with the S148A mutation resulted in a ∼16-fold decrease in the hydride transfer rate. Because the Met20 and F-G loops undergo a µs-ms chemical exchange event in the wild type and not in the mutant E:NADP+:FOLATE complexes, it was proposed that the exchange event is linked to the chemical step [6]. Our results show that the G121V mutation actually promotes a µs-ms exchange event involving the Met20 and F-G loops in the transition state analog complex that is absent in the wild type enzyme. This implies that for the case of the E:NADPH:MTX transition state analog complex, such µs-ms switching of these loops is actually anti-catalytic. This makes sense as precise positioning of multiple residues in the Met20 loop is required to orient the hydride donor and acceptor. Our data show that in the G121V transition state analog complex the F-G and Met-20 loops undergo excursions away from this proper alignment, which in our view, explains the reduction in *k*_hyd_.

## Materials and Methods

### Protein purification and NMR sample preparation

The G121V mutation was performed using the QuickChange Mutagenesis Protocol (Stratagene). Plasmid DNA was sequenced at the UNC Genomic Analysis Facility. Protein expression and purification was performed as described [22]. All NMR experiments were performed on 1 mM enzyme samples in buffer containing 70 mM HEPES pH 7.6, 20 mM KCl, 1 mM EDTA, 1 mM DTT, 20 mM NADPH, 3–5 mM MTX, 20 mM glucose 6-phosphate, and 10 U glucose-6-phosphate dehydrogenase. For the case of ^2^H relaxation, 2 mM enzyme and 40 mM NADPH were used. The E^G121V^ enzyme is saturated with cofactor and inhibitor based on the following: In spite of the modest NADPH binding defect reported [13], the cofactor is present at greater than 1000-fold over the *K*_D_ in the NMR samples. This translates to ≥99.9% saturation under the experimental conditions above. The wild-type E:NADPH complex has a picomolar affinity for MTX [36]. Because the G121V mutation has a negligible affect on binding of H2F and H4F [13], which are similar in structure to MTX and bind to the same site, MTX is also expected to bind G121V with very high affinity. Finally, all NMR samples were placed in amber NMR tubes and flame sealed under argon.

### NMR Experiments

All NMR experiments were performed at 298 K (calibrated with neat methanol) on Varian INOVA spectrometers. Backbone and side-chain methyl resonances were assigned using HNCACB/CBCA(CO)NH and HCCH_3_-TOCSY experiments, respectively, as described previously [43]. Tryptophan indole resonances were assigned using the ^15^N edited 1H NOESY. Standard backbone *R*_1_, *R*_2_ and [^1^H]-^15^N NOE [44] and side chain *D*_z_ and *D*_y_ [31], [32] relaxation spectra were collected as described previously [22]. Backbone relaxation was performed at 500- and 600 MHz whereas side-chain relaxation experiments were performed at 600- and 700 MHz. Backbone relaxation experiments were collected on 1 mM and 2 mM (600 MHz only) protein. The 2 mM sample was used to obtain τ_m_, which was required for fitting the methyl model-free data. Side-chain relaxation measurements were performed on 2 mM DHFR G121V. To identify enzyme regions with chemical exchange on the µs-ms timescale, relaxation compensated ^15^N CPMG experiments were performed essentially as described [22]. The constant time relaxation delay was set to 40 ms and three planes were collected: a reference plane with no relaxation delay and two CPMG planes with 1/τ_cp_ = 100 and 1800 s^−1^. Chemical exchange under our experimental conditions arises from *intra*molecular events rather than cofactor/drug binding and release. This is based on the fact that the enzyme is predicted to be ≥99.9% bound to both molecules (see above) and a free population of greater than 0.5% is typically required for measurable dispersion. Further, MTX binding is in slow exchange on the chemical shift timescale; thus a population of free enzyme would be manifest by additional resonances and we do not observe these. By contrast, the concentration of free NADPH would render overall exchange (*k*_ex_) due to binding and dissociation of cofactor too fast to yield dispersion using the CPMG fields employed here.

### Analysis of NMR dynamic parameters

Ps-ns backbone amide and side-chain methyl dynamics were characterized using the Lipari-Szabo model-free formalism [27], [28]. Consistent with wild type DHFR, the isotropic rotational correlation time for DHFR G121V is 10.7 ns/rad or 11.8 ns/rad for 1 mM or 2 mM protein, respectively. Rotational anisotropy was calculated using the local *Di* method [45] using the DHFR structure with PDBID 1RX3. The backbone relaxation data was fit using an anisotropic correction (D∥/D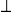 = 1.14) to minimize model selection error [46]. Backbone relaxation data were best-fitted to the five model-free models as described previously [22] using the in-house program relaxn2.2 assuming a 1.02 Å ^1^H-^15^N bond distance and −170 ppm ^15^N chemical shift anisotropy. For fitting tryptophan side-chain data, the chemical shift anisotropy of N^ε^-H^ε^ resonances was set at −89 ppm [47].
